# Diagnosis and laparoscopic management of retrocaval ureter: A review of the literature and our case series

**DOI:** 10.1016/j.ijscr.2019.05.036

**Published:** 2019-05-29

**Authors:** Maher Abdessater, Raghid El Khoury, Sandra Elias, Stephane Bart, Patrick Coloby, Walid Sleiman

**Affiliations:** a Centre hospitalier régional René DUBOS, Pontoise, France; b Centre hospitalier universitaire Notre Dame des Secours, Byblos, Lebanon

**Keywords:** Retrocaval ureter, Laparoscopic surgery, Literature review, Case series

## Abstract

**Objectives:**

To expose the diagnosis and the different laparoscopic approaches for the surgical management of patients with retrocaval ureter (RCU) and to share our experience on two cases.

**Methods:**

Updated literature review on Pubmed and debating personal experiences including ours (double j stent insertion before the surgery, use of 4 trocards, transperitoneal approach, pyelopyelostomy for the anastomosis…), concerning the laparoscopic treatment of the RCU.

**Results:**

Laparoscopic treatment of RCU is a recommended management for many reasons: less blood loss during the surgery, a shorter hospital stay, less postoperative pain and superior esthetic results with excellent functional results. All of these findings were also a part of our experience on the two reported cases: operative time was 210 and 180 min with no significant bleeding, hospital stay was 48 h post operatively for both patients that were symptom free with no renal dilation after 2 years of close follow up. The main cause of the increased operating time is the intracorporeal anastomosis of the ureter which remains the main limiting factor of the laparoscopic surgery.

**Conclusions:**

The literature review has clearly shown the advantages of minimally invasive techniques for the treatment of retrocaval ureter. Pure laparoscopic treatment (as in our two cases), seems feasible and technically reliable, and should be the standard surgical option for the treatment of RCU.

## Highlights

• The main advantage of minimally invasive techniques for the treatment of retrocaval ureter is less blood loss during surgery.
• Other advantages are shorter hospital stay, less postoperative pain and superior esthetic results.
• Pure laparoscopic treatment (as in our two cases) seems feasible and technically reliable with excellent functional outcome.
• Intracorporeal anastomosis of the ureter remains the main limiting factor.

## 1 Introduction

Variations in the anatomy of the vena cava or its tributaries can be encountered and if unrecognized can lead to life-threatening complications [1]. Retrocaval ureter (RCU), or circumcaval ureter, is a rare congenital disease which can cause obstruction and related symptoms. The first recorded case was seen on autopsy and described by Hochstetter in 1893 [2]. Since then, many surgical techniques were used to deal with this anatomical abnormality. Nowadays, mini invasive surgery is used for the correction of retrocaval ureter and the outcome has been excellent in all the described cases that we found in the literature till 2018 (Table 1).

**Table 1:** Summary of all reported cases of retrocaval ureter managed by mini invasive approach (PU = pyeloureteral, UU = ureteroureteral; NA = not available; PP = pyeloplasty, Lap = laparoscopic).

Year	Study	Number of cases	Approach	Anastomosis	Complications	Operative Time (min)	Follow-up (month)
1994	Baba et al. [36]	1	Laparoscopic transperitoneal	PU	No	560	2
1996	Matsuda et al. [38]	1	Laparoscopic transperitoneal	UU	No	450	Not reported
1997	Ishitoya et al. [16]	1	Laparoscopic transperitoneal	UU mini laparotomy	No	365	2
1998	Polascik et al. [44]	1	Laparoscopic transperitoneal	UU	No	225	4
1999	Salomon et al. [29]	1	Retroperitoneo-scopic	UU	No	270	6
1999	Mugiya et al. [39]	1	Retroperitoneoscopic	Automatic suture device	Not reported	300	Not reported
2001	Ameda et al. [34]	1	Transteritoneal	UU	No	450	3
		1	Retroperitoneal	UU	No	400	3
2001	Gupta et al. [32]	1	Retroperitoneal	UU	No	210	3
2003	Ramalingam et al. [37]	1	Transperitoneal	UU	No	240	7
		1	Transperitoneal	UU	Ileus	210	6
2003	Bhandarkar et al. [35]	1	Lap Transperitoneal	PP	NA	250	14
2005	Tobias-Machado et al. [41]	1	Lap Retroperitoneal	UU(extracorporeal)	No	130	3
2006	Simforoosh et al. [28]	3	Lap Transperitoneal	PP	No	180 (150–210)	3
		3					
2006	Nagraj et al. [45]	1	Lap Transperitoneal	UU	No	100	Not reported
2006	Guntedi et al. [43]	1	Robotic Transperitoneal	UU	No	180	6
2008	Chung and Gill [46]	1	Lap Transperitoneal	PP	No	180	6
2008	Hemal et al. [42]	1	Robotic transperitoneal	PU	No	Not reported	3
2009	Bagheri et al. [58]	3	Lap Transperitoneal	UU	No	210 (mean)	12
2009	Xu et al. [47]	7	Retroperitoneoscopic	UU	No	128.6 (97–189)	12
2010	Autorino et al. [48]	1	Trasperitoneal LESS	UU	No	180	3
2012	Nayak et al. [49]	5	Robotic transperitoneal	UU and PU	No	92 (mean)	13.5 (mean)
2012	Alkhudair et al. [50]	1	Robotic transperitoneal	UU	No	90	3
2012	Ding et al. [51]	6	Lap Transpertitonal	UU	No	163 (145–178)	3–30
2013	Junior et al. [52]	1	Lap Transperitoneal	PU	No	210	6
2014	Ji et al. [53]	18	8 Lap Trans	UU	No	Trans:85 ± 20	40 ± 24
			10 Lap Retro			Retro :98 ± 30	
2015	Ricciardulli et al. [54]	27	Lap Transperitoneal	UU	4 cases	131 (mean)	3, 6 and 12
2016	El Harrech et al. [55]	3	Lap Transperitoneal	PP	No	140 (110–190)	Every 3
2016	Fidalgo et al. [56]	1	Lap Transperitoneal	PU	No	170	3
2017	Lijun Mao et al. [57]	6	Lap Retroperitoneal	PU	No	112 (median)	6–24

In this article, which has been reported in line with the PROCESS criteria [3], we describe 2 cases of retrocaval ureter treated by laparoscopic transperitoneal pyelopyelostomy, with a review of the literature on the diagnosis and laparoscopic treatment of retrocaval ureter.

## 2 Case presentations

### 2.1 Case 1

**Fig. 1:**
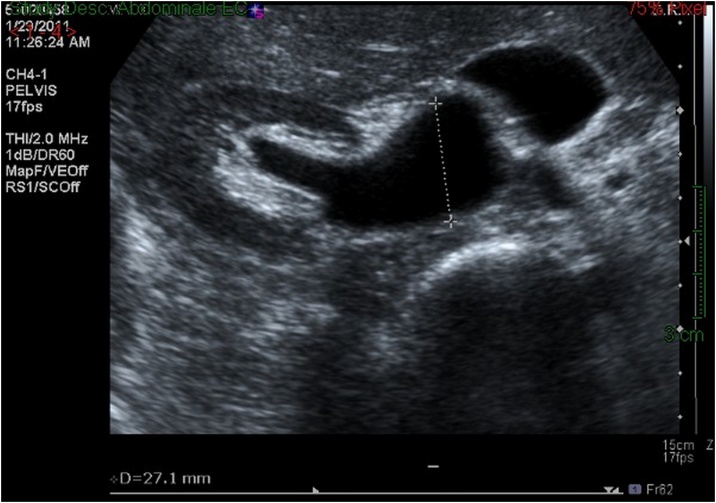
Renal ultrasound of patient 1 showing severe right-sided hydronephrosis.

**Fig. 2:**
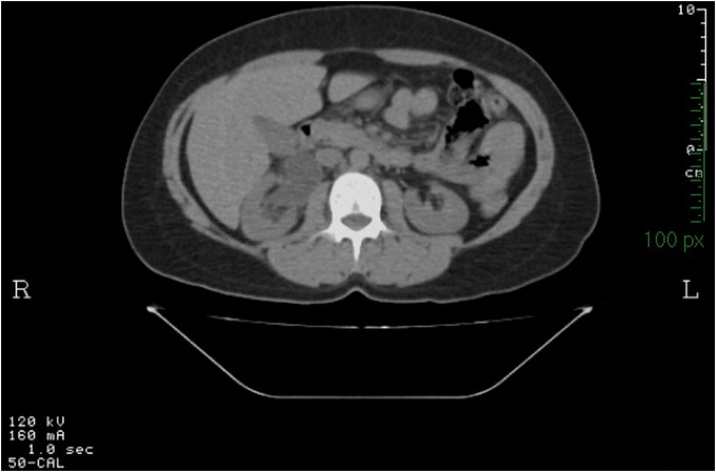
KUB scan of patient 1 suggesting the presence of a RCU.

A 43-year-old previously healthy woman presented for right-sided flank pain, fever, chills with leukocytosis, history of UTI treated by antibiotics 2 weeks ago, with long history of recurrent intermittent right-sided flank pain not investigated. Renal ultrasound showed severe right-sided hydronephrosis (Fig. 1) and computed tomography scan suggested the presence of a RCU (Fig. 2).

**Fig. 3:**
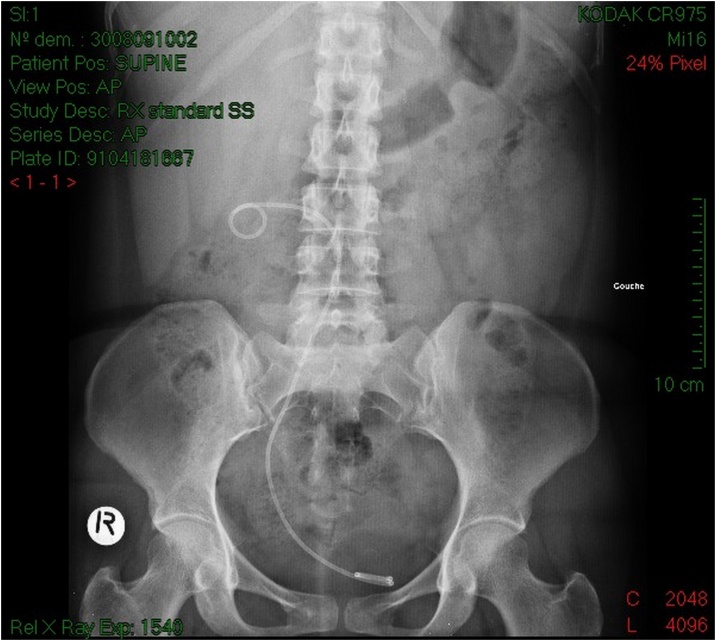
KUB of patient 1 showing the double J stent that takes the S shape of the ureter.

**Fig. 4:**
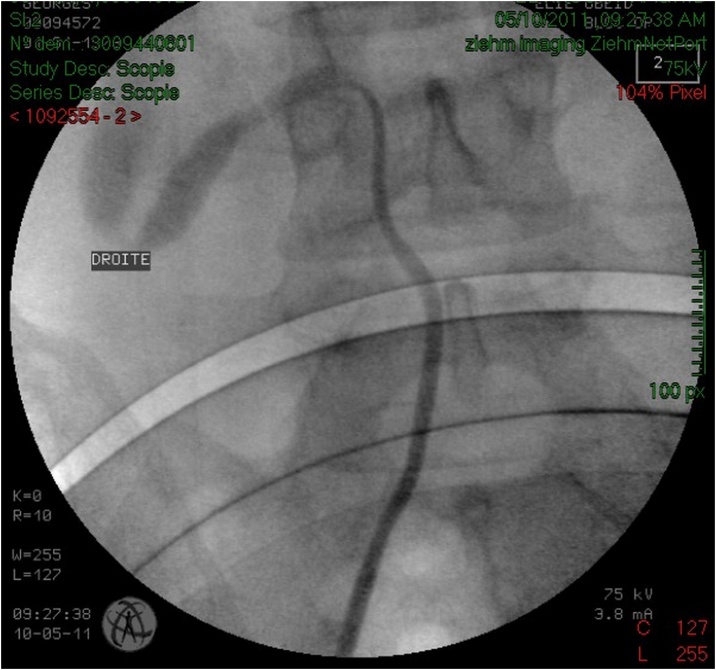
Retrograde ureteropyelography of patient 1 confirming the diagnosis of retrocaval ureter.

Serum testing demonstrated preserved renal function, urine culture turned positive for proteus mirabilis. The patient was treated with intravenous antibiotics and drainage of the right kidney by a double J stent (Fig. 3) with retrograde ureteropyelography that confirmed the diagnosis of retrocaval ureter (Fig. 4), antibiotic treatment continued for 2 weeks orally, and laparoscopic repair of retrocaval ureter was planned after sterilization of the urine.

### 2.2 Case 2

**Fig. 5:**
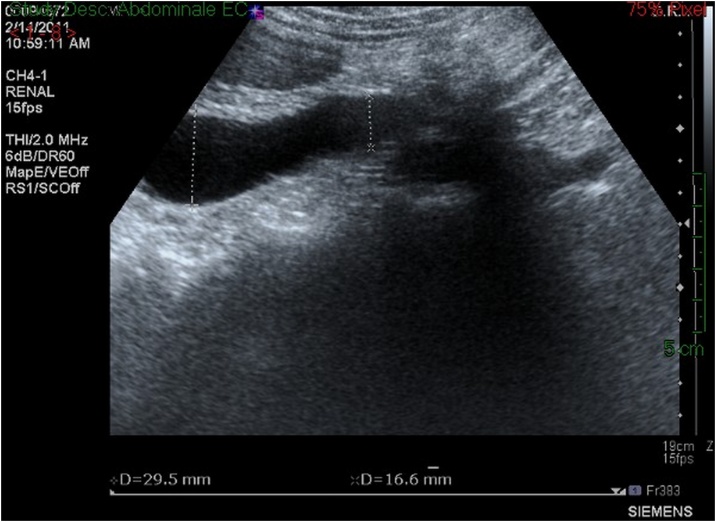
Renal ultrasound of patient 2 showing severe right-sided hydronephrosis.

**Fig. 6:**
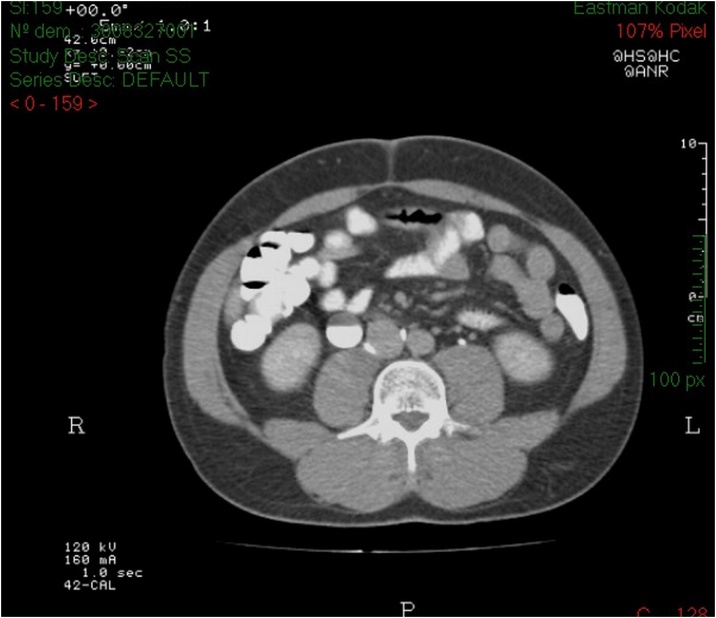
Computed tomography scan of patient 2 showing the presence of a retrocaval ureter.

**Fig. 7:**
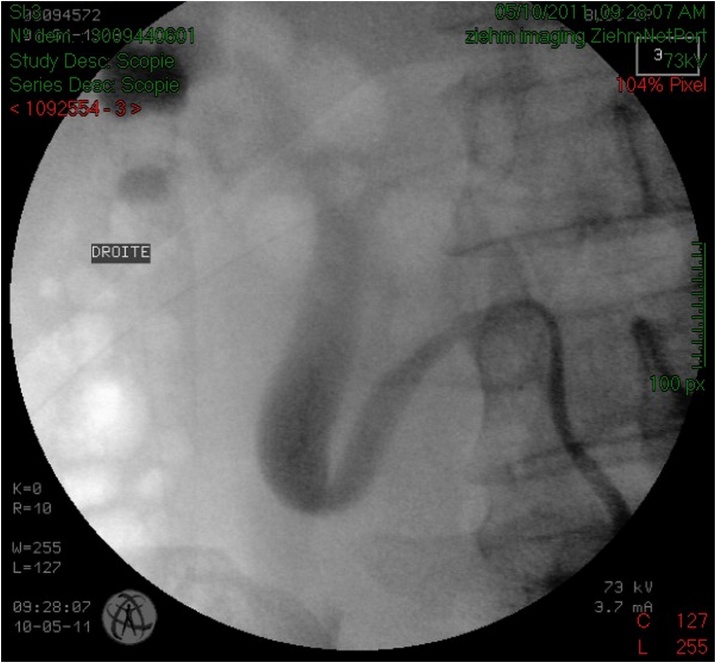
Right retrograde ureteropyelography of patient 2 showing the S shape of the ureter.

**Fig. 8:**
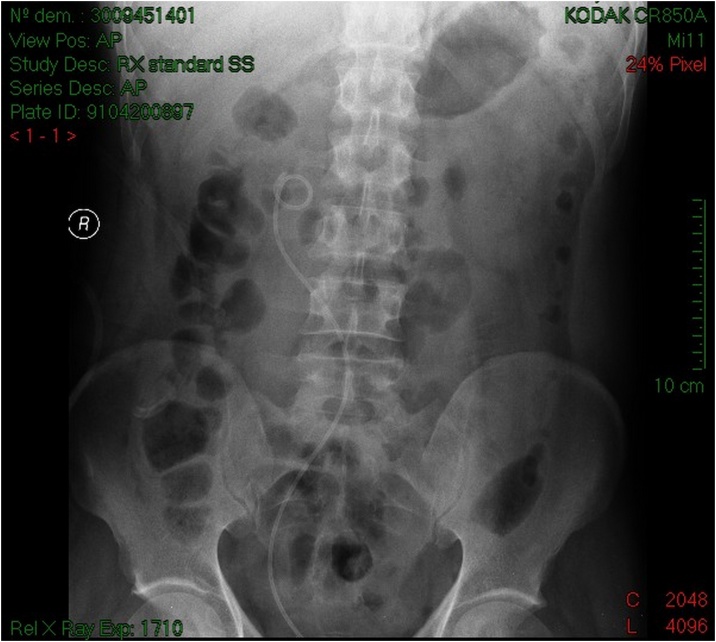
KUB of patient 2 showing the double J stent that takes the S shape of the retrocaval ureter.

A 38-year-old man previously healthy presented for a recurrent intermittent right-sided flank pain exacerbating since about 1 year ago, aggravated by water intake and associated with irritative (urinary frequency) lower urinary tract symptoms (LUTS), history of left renal colic with spontaneous passage of small stone about 3 years ago not investigated. Renal ultrasound demonstrated sever right-sided hydronephrosis (Fig. 5) and computed tomography scan suggested the presence of a retrocaval ureter (Fig. 6), Serum testing demonstrated preserved renal function, and a laparoscopic repair of retrocaval ureter was planned, with a right retrograde ureteropyelography (Fig. 7), done just before the surgery for a retrograde double J stent placement (Fig. 8), and confirmed the diagnosis of retrocaval ureter.

### 2.3 Surgical technique

**Fig. 9:**
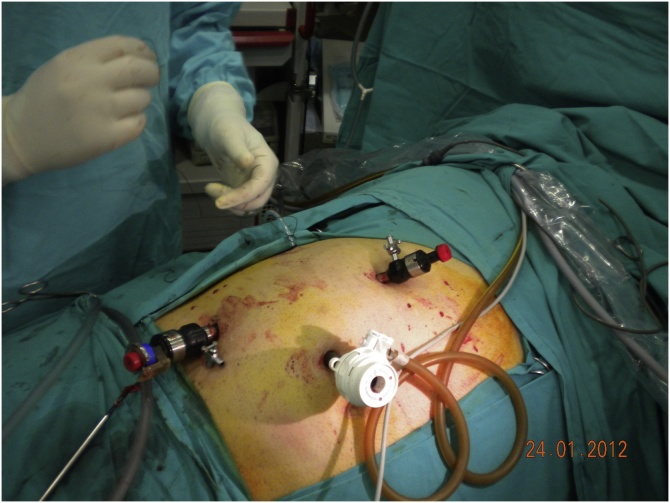
Trocars’ positions (same laparoscopic technique for the two patients).

**Fig. 10:**
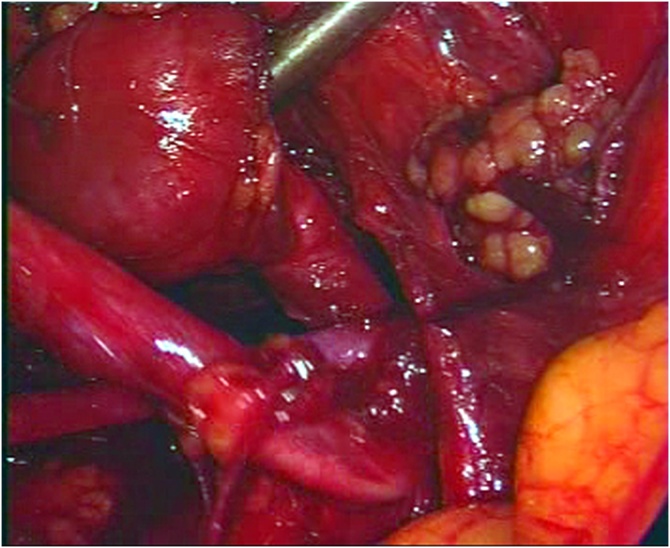
Dilated proximal renal pelvis and ureter were found and separated from the adjacent tissue by a blunt and sharp dissection.

**Fig. 11:**
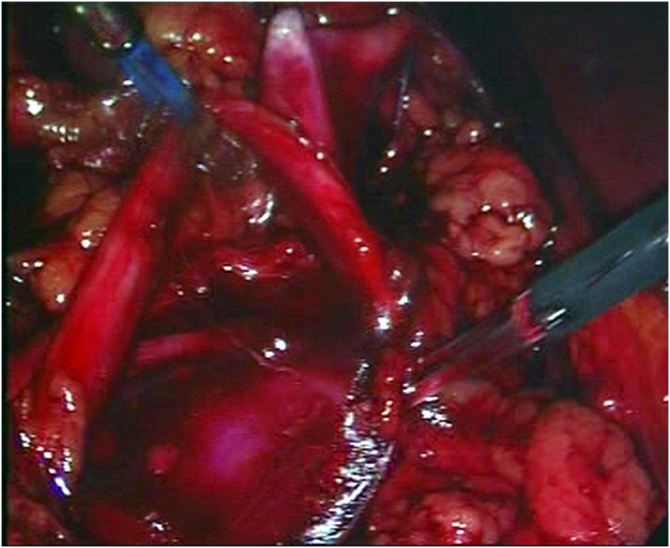
Transection at the ureteropelvic junction.

After induction of general endotracheal anesthesia an orogastric tube and a Foley catheter were inserted. The patient was positioned in a 60-degree left lateral decubitis position and a transperitoneal approach was used. A pneumoperitoneum was created using a Veress needle and ports were entered applying a 20 mm Hg intra-abdominal pressure. First, a 10-mm camera port was placed at the umbilicus level on the lateral rectus border. After the placement of the first port, the others were placed under direct vision. A 10 mm working port was placed 1 cm below the costal border on the midclavicular line, and a 5 mm port at a point on the lateral one-third of the line between the anterior superior iliac spine and the umbilicus. The 5-mm fourth trocar was placed for traction on midaxillary line at the level of the umbilicus (Fig. 9). The intracorporeal pressure was decreased to 12 mm Hg after the placement of the ports. After the incision of the Toldt line, the dilated proximal renal pelvis and ureter were found and separated from the adjacent tissue by a blunt and sharp dissection (Fig. 10). Traction of the proximal ureter by a stich of the renal pelvis through the abdominal wall, helped to identify the distal ureteral segment which was released without damaging the periureteral soft tissue. After the complete release of the ureteral segment beneath the inferior vena cava, the dilated renal pelvis was transected at the ureteropelvic junction (Fig. 11). The ureter was separated from the inferior vena cava and positioned anterior to it. The dilated renal pelvis was anastomosed (pyelopyelostomy) with 4-0 vicryl intracorporeal sutures inserted in a continuous manner posteriorly and interrupted sutures anteriorly after the introduction of the proximal curve of the double J stent inserted before the surgery. After the completion of anastomosis, a closed suction drain was placed in the operation area.

### 2.4 Results

The operative time was 210 and 180 min for patients 1 and 2 respectively. Neither intraoperative complication nor significant bleeding occurred. Urethral catheters were removed 24 h post surgery, the drains were removed 1 day after catheter removal, and the patients were discharged 48 h after the operation after return of bowel function. No significant dilatation was observed on ultrasound at the fourth week follow-up as well as after 3, 6 12 and 24 months. No patient had problems in the early postoperative period. Post-surgery, the patients were followed up with ultrasound at the fourth week and JJ stents were removed at the sixth week. At 3-month follow-up, diuretic radionuclide scan revealed no evidence of obstruction of the right kidney, and the excretory urogram revealed prompt excretion of the contrast material, with no dilatation of the repaired renal unit and a widely patent anastomosis in both patients.

Within the follow-up period patients were tracked by ultrasound at the 3rd, 6th, 12th and 24th month and questioned for the presence of postoperative symptoms. The two patients showed improvement in dilatation and had no symptoms.

## 3 Review of the literature

### 3.1 Pathologic embryology

**Fig. 12:**
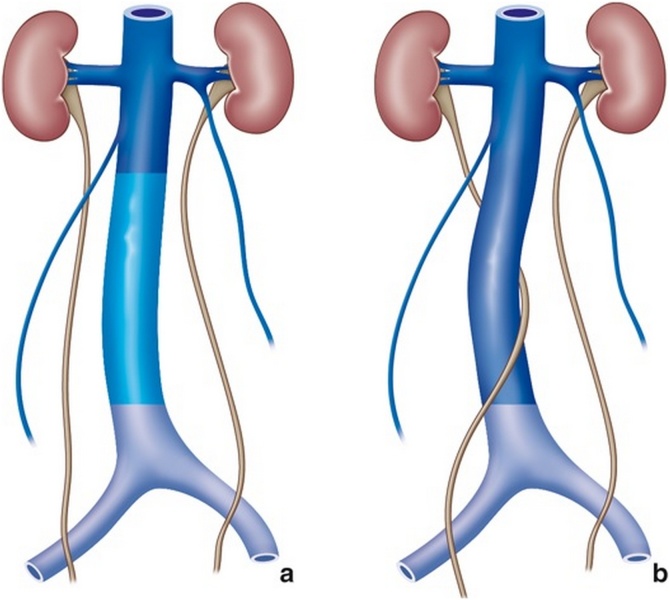
Persistence of the posterior cardinal vein ventral to the ureter entrapping it.

Because of the persistence of the posterior cardinal vein that is located ventral to the ureter, the ureter will become entrapped behind the vein (Fig. 12). Thus, the ureter, predominantly the right ureter, courses behind the IVC, then between the vena cava and aorta, and continues to course downward anterior and, finally, lateral to the IVC.

### 3.2 Epidemiology

The incidence has been reported as 1 in 1000 individuals [4,5].

This anomaly is almost exclusively found on the right; however, 1 case of a left-sided retrocaval ureter in association with situs inversus and 1 case of bilateral involvement [6,7] have been reported, it usually manifest in the third or fourth decade of life [8], and occurs tree more times frequently in men than in women [9].

### 3.3 Classification

**Fig. 13:**
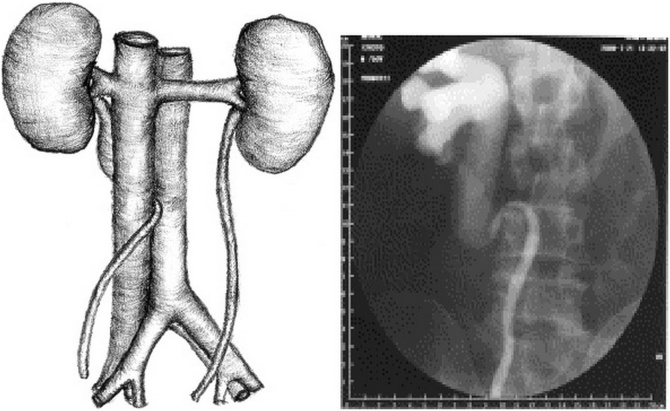
RCU Type I (low loop) with the dilated proximal ureter assuming the shape of a reverse “J”.

In 1982, Bergman classified retrocaval ureter into two clinical types [10]. Type I (low loop) (Fig. 13) is the most common, with the dilated proximal ureter assuming the shape of a reverse “J”. Usually, this type of ureter is obstructed. Type II (high loop) is seen less frequently. The ureter passes behind the IVC at the level of, or just above, the pelvic-ureteral junction. This type of ureter is frequently not obstructed.

### 3.4 Associated congenital anomalies

Approximately 20% of the patients with retrocaval ureter may had accompanying congenital abnormalities including solitary kidney, contralateral renal hypoplasia or ectopia [11], horseshoe kidney [12,13], aberrant renal artery [14], Turner’s syndrome [15], Goldenhar syndrome [16], retroperitoneal fibrosis [17], and polycystic disease of the kidneys [18], nutcracker symdrome, ureterocele, ureteropelvic junction obstruction [19,20] genital malformation (hypospadias), duplicated IVC, situs inversus, intestinal malrotation, cardiovascular anomalies (coarctation of the aorta, pulmonary venous stenosis) [21], and myelomeningocele [22].

### 3.5 Clinical aspect

About 80% of cases are symptomatic, with no pathognomonic symptom for the RCU: Right flank pain being the most common symptom occurring in about 70% of cases, varying from a dull, persistent and well localized pain to severe renal colic according to the nature and degree of the ureteral obstruction, associated sometimes with atypical abdominal or pelvic pain, and vomiting. Hematuria either micro or gross hematuria may be present in about 20% of cases, can be isolated or associated with flank pain is related to urolithiasis in most cases.

Infection of the upper urinary tract (acute pyelonephritis) with fever, leucocyturia, bacteriuria, sometimes pyonephrosis and bacteremia may be present in about 20% of cases and should prompt urgent diagnostic and therapeutic management [23].

### 3.6 Diagnostic imaging

Ultrasound contributes to diagnosis because it highlights pyelo-calyceal dilatation of the right kidney signing the obstacle on the urinary tract [24]. The CT Urography is now the method of choice for diagnosing retrocaval ureter; it is the most efficacious and least invasive method of confirming the diagnosis, it allows the differential diagnosis of ureteral obstructions due to an acquired retroperitoneal pathology, especially malignant tumors and retroperitoneal fibrosis. Compared to CT Scan, MRI is equally good, has no radiation risk, and does not require an iodinated contrast. Renal scan can assess the degree of obstruction (DTPA), as well as the renal function (DMSA), and may be used for the follow up of asymptomatic patients with retrocaval ureter [[25], [26], [27]].

### 3.7 Conservative surgical treatment

Despite active surveillance in asymptomatic patients with normal renal scan and nephrectomy for patients with destroyed kidney, conservative surgical treatment is indicated for all symptomatic patients with preserved ipsilateral renal function.

The principle of conservative surgery for retrocaval ureter is the restoration of normal anatomic position by transpositioning of the ureter over the inferior vena cava. This can be done by open surgery, laparoscopic transperitoneal [28], laparoscopic retroperitoneal [29], as well as by robotic assisted surgery [30].

Conventionally, open access has been considered the standard procedure for the correction of RCU [31]. However, in the last decade, with the intensive growth of minimally invasive surgery the laparoscopic approach has proved to yield equivalent therapeutic results, with superior aesthetic outcomes, fewer analgesic requirements, a shorter hospital stay [32,33] and more rapid convalescence. The limiting factors are the learning curve of laparoscopic operations and the difficulty in intracorporeal suture techniques [34,35], both of which can be overcome if such patients are referred to urologic centers with laparoscopic experts. New advances in laparoscopic suturing techniques have improved dramatically in the past few years.

Table 1 resumes the different results published in the articles on the minimally invasive surgical management of the RCU from 1994 till now, it was done after a thorough literature review on Pub Med:

In 1994, Baba was the first to perform a laparoscopic pyelopyelostomy using a transperitoneal approach for retrocaval ureter [36].

Salomon et al. [29] reported the first case of retroperitoneoscopic repair of retrocaval ureter in a young man. Gupta et al. [32] used a three-port retroperitoneoscopic approach to perform ureterolysis and ureteroureteral reconstruction that lasted for 3.5 h.

The decision of whether to resect or preserve the retrocaval segment of the ureter has been controversial. Simforoosh et al. [32] reported 6 cases without excision of the retrocaval segment. They transected at the level of the dilated renal pelvis and performed pyeloplasty, with successful results. However, in all their patients, the retrocaval segment appeared grossly normal [32]. Zhang et al. [35] suggested excising the retrocaval segment if an 8F catheter could not pass through the segment easily. We think that the radiologic findings and intraoperative appearance of the ureter should be used to decide whether or not to excise the retrocaval segment. Its preservation should be considered only if this segment has had a normal appearance, without considerable kinking. In addition, with each ureteral peristalsis, the upper ureter should not dilate further and the peristaltic movement should be seen throughout the entire length of the retrocaval segment. In such cases, it is preferred to transect the ureter at the lower part of the dilated ureter, lateral to the IVC. This makes the anastomosis easier at a site with an ample blood supply and decreases the probability of stricture formation. However, if any of these findings are missing or doubt is present, one should transect the ureter medial to the IVC and resect the upper stump until a non-kinking dilated ureter and urine flow are clearly detected.

We have performed ureteroureteral or ureteropelvic end-to-end anastomosis, depending on the level of ureteral transaction, with approximately 1-cm spatulation of the lower ureteral stump and, in selected cases, of the upper ureteral stump is recommended. Insufficient spatulation can result in stricture formation at the site of anastomosis.

The choice of either a transperitoneal or a retroperitoneal approach depends on surgeon preference. There were different results in the transabdominal and retroperitoneal laparoscopic repair of the retrocaval ureter. Ramalingam and Selvarajan [37] presented their experience with transperitoneal LUUS of retrocaval ureter in 2 cases. They believed that transperitoneal intracorporeal suturing is less time-consuming and easier than retroperitoneoscopic suturing. Gupta et al. [36] found retroperitoneoscopic approach to be safer, easier, and less time-consuming, and it provided direct access to the ureter and IVC while avoiding spillage of urine into the peritoneal cavity. Mugiya et al. [43] confirmed that the retroperitoneoscopic treatment could be superior to the conventional transabdominal approach to perform the laparoscopic transposition and re-anastomosis of a retrocaval ureter, because it provides a shorter and more direct access to the ureter, without interference from intra-abdominal structures. Although the working space for laparoscopic manipulation is relatively limited, results have clearly showed that it is effective when an experienced surgeon performs reconstructive procedures for this approach. Gupta et al. [32] and Salomon et al. [29] suggested that the retroperitoneal laparoscopy represented the more direct approach to the urinary tract and the shorter time was obtained because dissection of the retroperitoneal space was not hindered by intra-abdominal organs.

The approach performed frequently is the transperitoneal approach because of a larger operation field and because of urologists who are more familiar with this approach [37,38]. The retroperitoneal approach was also described earlier because of the concerns of urine leaking into the peritoneum [39,40]. However, the surgical field is narrower in this approach, and we believe that a transperitoneal watertight anastomosis over an internal stent is straightforward and does not pose any postoperative problems. Another technique using laparoscopically-assisted extracorporeal anastomosis through a 2 cm skin incision has been described with the aim of decreasing the operative time [41]. However, this approach is not compatible with the aim and principle of laparoscopy.

The transumbilical single-port (LESS technique) approach is practical and affords a virtually scar-free result. Experience is increasing, and the first successful case of LESS repair of retrocaval ureter using the GelPoint access platform reported by Robert J. Stein et al. from the Center for Laparoscopy and Robotic Surgery, Glickman Urological and Kidney Institute, Cleveland Clinic. The procedure was technically successful and operative time at 3 h was comparable to that of reported standard laparoscopic series. LESS procedures remain somewhat cumbersome, and further refinement in instrumentation is needed before the technique may achieve the level of standardization.

Given the advances in robotic-assisted laparoscopic surgery, RCU which has been operated using pure robotic laparoscopy was reported [42,43]. The authors stated that robotic assistance eases the dissection and intracorporeal suturing and knotting. However, it is not available in every institute. Recognized benefits of the da Vinci robotic System over conventional laparoscopy include superior ergonomics, 3-dimensional optical magnification of the operative field within direct control of the console surgeon, enhanced surgeon dexterity within the field of view, and precision of surgical manipulation with tremor reduction.

Finally, for the operative time, we believe that the running re-anastomosis of the transected renal pelvis (eliminating much intracorporeal knot tying) and intraoperative placement of a Double-J^®^ stent (thus, forgoing preliminary cystoscopy, guide wire or stent placement and patient repositioning) contributed significantly to a shorter operative time as for example in the series of Simforoosh et al. [28].

## 4 Conclusion

Laparoscopic treatment of retrocaval ureter is a recommended management for many reasons: less blood loss during the surgery, a shorter hospital stay, less postoperative pain and superior esthetic effect with excellent functional results. All of these findings were also a part of our experience on the two reported cases. The main cause of the increased operating time is the intracorporeal anastomosis of the ureter which remains the main limiting factor of the laparoscopic surgery.

The literature review has clearly shown the advantages of minimally invasive approaches.

Pure laparoscopic treatment of RCU seems feasible and technically reliable, and should be the standard surgical option for the treatment of RCU.

The laparoscopic magnification provides an excellent exposure, allowing an adequate dissection of ureter in situ. The retrocaval segment could easily be freed from the vena cava. Adequate dissection and mobilization of the ureter and periureteral tissue is mandatory to ensure a tension-free anastomosis while preserving its blood supply. Unnecessary dissection should be avoided to obtain the best possible results.

## Conflicts of interest

No conflict of interest.

## Funding

No sponsor for this article.

## Ethical approval

This work is exempt from ethical approval in our institution because of its type.

## Consent

Written informed consent was obtained from the patients for publication of these case reports and accompanying images. A copy of the written consents is available for review by the Editor-in-Chief of this journal on request.

## Author contribution

EL KHOURY, SLEIMAN, ELIAS and ABDESSATER conceived of the presented idea and were encouraged by COLOBY to execute it.

BART, ABDESSATER, EL KHOURY and SLEIMAN were on the operating field while performing the surgeries and all of them participated to the different steps of the surgery but EL KHOURY was the main surgeon.

SLEIMAN, BART, ELIAS and ABESSATER chose and cropped the most important figures from the surgery’s video and pathology.

BART, SLEIMAN and EL KHOURY contributed to the final version of the manuscript. COLOBY supervised the work.

ABDESSATER and ELIAS took the lead in writing the manuscript when they found that It deserves to be published.

All authors provided critical feedback and helped shape the manuscript.

The 6 authors designed the model and the computational framework and analyzed the results.

All persons who meet authorship criteria are listed as authors, and all authors certify that they have participated sufficiently in the work to take public responsibility for the content.

## Registration of research studies

Research registry 4708.

## Guarantor

Patrick COLOBY and Walid SLEIMAN are the guarantors of this work.

## Provenance and peer review

Not commissioned, externally peer-reviewed.
